# The Meaning of Critical Illness for People Suffering From COVID-19: When a Frightening Unreality Becomes Reality

**DOI:** 10.1177/10497323211050048

**Published:** 2021-11-27

**Authors:** Åsa Engström, Päivi Juuso, Maria Andersson, Anna Nordin, Ulrica Strömbäck

**Affiliations:** 1 Department of Health, Education and Technology, Division of Nursing and Medical Technology, 91874 Lulea University of Technology, Luleå, Sweden; 2 Faculty of Health, Science, and Technology, Department of Health Science, 101086 Karlstad University, Karlstad, Sweden

**Keywords:** COVID-19, critical illness, nursing, qualitative, philosophical hermeneutic interpretation

## Abstract

The aim of this study was to elucidate the meaning of critical illness for people with COVID-19. This study used a qualitative design. Thirteen people who were critically ill with COVID-19 during 2020 and admitted to a COVID-19 intensive care unit in northern Sweden participated in the study. Data collection was conducted as individual interviews with a narrative approach, and data were analyzed with phenomenological hermeneutic interpretation. The participants did not think they would get critically ill with this unexpected illness. They experienced terrible nightmares where their relatives had been killed, and they missed their relatives both in their dreams and in reality, as they had not been allowed to be with them due to the virus. Gratefulness was described for surviving. Participants described thoughts of not being able to imagine going through this again. They felt fear and loneliness, as a terrifying unreality had become a reality.

## Introduction

The COVID-19 pandemic has placed enormous pressure on healthcare systems around the world. Millions of people have become critically ill and required care in intensive care units (ICUs) (Simpson & Robinson, 2020). The most frequently reported reason for admission to intensive care has been the need for mechanical ventilation (Wang et al., 2020). From the patients’ point of view, Engström et al. (2013) conclude that the experience of being mechanically ventilated in an ICU creates a sense of being delivered into the hands of others, as the person being ventilated cannot trust their body to function. Being connected to tubes and unable to breathe or communicate is stressful, which is in line with studies about being critically ill and mechanically ventilated (Cypress, 2011; Engström et al., 2013). In an integrative review, Aitken et al. (2016) show that there is limited evidence about the association between elements of ICU treatment and memories after ICU discharge. They found that deep sedation, corticoids, and administration of glucose 50% due to hypoglycemia probably contribute to the development of delusional memories and amnesia regarding the ICU stay. The inability to distinguish real events from hallucinations is commonly described (Cutler et al., 2013). The presence of relatives during and after critical illness is vital for the person who is ill (Engström & Söderberg, 2007; Engström, 2008), as they are a link to the patient’s everyday life and can fill in lacking time. Several studies (Engström, 2008; Engström et al., 2015; Löf et al., 2010) show the importance of a critically ill person’s close relatives being near and taking part in what is happening with the ill person. Before the COVID-19 pandemic, a patient’s relatives were welcomed to be with the critically ill person in the ICU, which was seen as doing good for the ill person, relatives, and ICU staff (Engström, 2008). Unfortunately, during the COVID-19 pandemic, relatives have not, in most cases, been permitted or able to visit the critically ill patient with COVID-19 due to the highly contagious nature of the disease.

Nursing critically ill patients suffering from COVID-19 is similar to the intensive care of patients with similar diagnoses, such as respiratory failure and need for mechanical ventilation (Domínguez-Cherit et al., 2009), but at the same time, something completely different. The similarities are the given nursing care, treatments, and observations made. The differences are the environment where the patients are cared for and challenges for teams working together under the specific circumstances of nursing patients with COVID-19, for instance, the decreased possibilities to communicate adequately. According to Cook et al. (2020), the management of patients with known or suspected COVID-19 requires specific safety considerations for staff, patients, and relatives, which means protection from the virus. The nursing workload during the COVID-19 pandemic has dramatically increased (Fernández-Castillo et al., 2021; Lucchini et al., 2020). It is considered that ventilation in the prone position, if needed, should be initiated early and maintained for 14–16 hours a day for at least 3 days (Pedersen et al., 2020). In addition, with COVID-19 patients, healthcare workers have an elevated risk of exposure, and the use of personal protective equipment (PPE) is mandatory.

Although several studies (Feeley et al., 2021; San Juan et al., 2021) focus on the staff perspective of the intensive care for patients with COVID-19, there is a lack of studies about the experience of being critically ill due to COVID-19. Common reasons for admission to the ICU due to COVID-19 during spring 2020 were hypoxemic respiratory failure leading to mechanical ventilation, hypotension requiring vasopressor treatment, or both (Bhatraju et al., 2020). Recent studies (Marshall, 2020; Salehi et al., 2020) have also raised the question regarding long-term consequences after COVID-19, affecting other parts than the lungs, impacting on the daily life also after recovery. We need to know more about what this means to people who have been critically ill, and we need to be prepared for similar pandemics. Therefore, the aim of this study was to elucidate the meaning of critical illness for people with COVID-19.

### Design

This study used a qualitative design. Data collection was conducted as individual interviews with a narrative approach, and data were analyzed with phenomenological hermeneutic interpretation.

### Setting and Procedure

This study was conducted in hospitals in northern Sweden with COVID-19 ICUs. The hospitals in the region responded to the pandemic, reorganized, and established special COVID-19 ICUs to provide intensive care for these patients. Most patients were transferred from other hospitals in the region and were intubated and invasively (mechanically) ventilated upon admission to the ICU. The mode of ventilation was pressure-controlled ventilation, tidal volume 6 mL/kg, positive end-expiratory pressure (PEEP) 12-18 H2O, and a high level of Fi O2, that is, ≥ 50%, due to the gravity of their critical illness. The initial regime for sedation and analgesia was midazolam, morphine, clonidine, and continuous infusion of rocuronium. A majority of the patients were ventilated in the prone position about three to four times during the first phase of the intensive care. The prone position lasted for at least 18 hours, followed by 8 hours on the back. In half of the patients, a tracheostomy was performed when it was no longer indicated to ventilate the patient in the prone position. At that time, the regime for sedation and analgesia changed to propofol, remifentanil, and/or dexmedetomidine.

### Ethical Considerations

The head of the ICU provided written permission to conduct the project. The ethical committee in Sweden approved the study (nr 2020-02805). All participants verified their participation by filling in and returning a written consent. Before the interviews, all participants received verbal and written information about the nature of the study. They were reassured that their participation was voluntary and that they could withdraw from the study at any time without giving any explanation. As each interview began, Åsa Engström checked that the participant understood the aim of the study. The participants were guaranteed confidentiality and an anonymous presentation of the findings.

### Participants and Data Collection

Seventeen people who were critically ill with COVID-19 during the spring of 2020 and admitted to one COVID-19 ICU in northern Sweden were informed about the study and asked by mail to participate in the study using purposive sampling. Thirteen agreed to participate by sending a written signed consent. The age of the participants ranged from 32 to 78 years (mean = 60,15, SD 12,24); eight participants were male, and five were female. They had been intubated/received tracheostomy and received respiratory treatment due to COVID-19 for 9–56 days (md = 20). The interviews took place from July 2020 to March 2021. Data were collected by Åsa Engström using individual telephone interviews (*n* = 10) and face-to-face interviews (*n* = 3). The participants were asked to describe their experiences with COVID-19, becoming critically ill, and receiving intensive care due to COVID-19. Follow-up questions were asked, such as: What happened then? How did you feel? Can you give an example? The interviews lasted between 35–80 (md = 55) minutes, were audio-taped and transcribed verbatim.

### Data Analysis

#### Phenomenological Hermeneutics Interpretation

Drawing from phenomenological hermeneutics, the interview transcripts were analyzed to interpret them according to a dialectical process that prioritized understanding and explanation (Ricoeur, 1976). According to Ricoeur (1976), the relationship between phenomenology and the hermeneutic philosophy can be assessed to discover the meaning of lived experiences. The interpretation of the transcripts involved three stepwise phases: the formation of a naïve understanding, structural analysis, and the formation of a comprehensive understanding (Lindseth & Norberg, 2004). In the first step, the transcripts were read and reread several times to grasp the overall meaning of the experiences related and, in turn, form a naïve understanding of those experiences. Second, during structural analysis, the transcripts were de-contextualized (Lindseth & Norberg, 2004), which involved dividing the transcribed text into units of meaning, later organized into sub-themes and, in turn, a primary theme. The sub-themes and theme were deliberated to validate or invalidate the naïve understanding, under the assumption that the trustworthiness of structural analysis depends on the coherence of the parts and the whole. Throughout the analysis, the perspective taken continually alternated focus between the individual transcripts, the transcripts as a whole, and the interpretation of them. The interpretation presented here is the one we found to be the most credible understanding of the text. According to Ricoeur (1976, p.76) “*an interpretation must not only be probable, but more probable than another interpretation.*” We were aware of our pre-understanding as specialist nurses and as nurse researchers, and tried to be as open as possible to the text by continually and critically reflecting on the interpretation. Third, to form a comprehensive understanding, the naïve understanding and structural analysis results were integrated to afford an in-depth interpretation. The comprehensive understanding of the related experiences as a whole took shape with reference to the authors’ preconceptions, the aim of the study, and literature on the topic.

## Findings

### Phenomenology Hermeneutics Interpretation

#### Naïve Understanding

None of the participants expected to be affected by COVID-19 and first realized its seriousness after the virus affected people close by. Still, it seemed to be more evident when they themselves became critically ill, for which they felt totally unprepared. During the illness, feelings of fear were salient, frightening events that felt real, such as one’s organs being sold or one’s relatives being killed, which gave horror. It took a while to realize what had happened and that these horrible experiences were not real. Being dependent on others for managing own hygiene led to feelings of being undignified. Simultaneously, feelings of relief were evident for the support they got as they felt extraordinarily weak and lacked the strength to manage independently. During the time of isolation, feelings of loneliness were common, despite having healthcare professionals around them. The contact and reunion with relatives were experienced as joyful. Feelings of gratitude for having survived COVID-19 were palpable, which seemed to increase the joy for small things in life, things previously taken for granted. Feelings of being more emotional were described along with an uncertainty about being able to go through something similar again since the experience had been so tough.

### Structural Analysis

The structural analysis resulted in one overall theme with three sub-themes (Table 1)

**Table 1:** Overview of the Findings Presented as Theme (*n* = 1) and Sub-Themes (*n* = 3).

Theme	Sub-themes
When a frightening unreality becomes reality	A battle against an unknown enemy
	A living nightmare
	Feeling weak and lonely

### A Battle Against an Unknown Enemy

The participants described that when they first heard about COVID-19, they found it to be a bit scary but did not think it would reach the northern parts of Sweden or affect them. When the virus eventually came to Sweden, they experienced the information as exaggerated. Their experience from the information was that it was mainly dangerous for older people, and they were afraid for their parents’ sake. Participants described that they first realized the seriousness when they also became ill and in need of intensive care due to COVID-19.

*I have to say I was a little naive. I didn’t think it would affect us. That it would be like, well, what should I say, the misery of it all. But the closer it got, the more I started to realize that this was something else.*

*The general feeling in the beginning was that it was a flu, and we thought as overstated it was nonsense. Then it turned out that we had quite a lot of people here (at work); it was a little strange time because half of them were very anxious and the other half thought it was nonsense. I was one of those who thought it was nonsense, and then I discovered that it was not so.*

The participants described that the illness started like the flu, with high fever, sore throat, weakness, breathing difficulties, stomach problems, and being delirious. At this point, they needed to be tested for COVID-19; they described the testing as a long wait outside the healthcare center or hospital, which was experienced as exhausting, as they felt weak. The course of the disease was described as unpredictable, as they initially became quite ill and then started to feel better, which gave them the sense that they were on the road to recovery. During this illness period, they were at home, isolated with someone within their family, or alone. They described trying to manage easier tasks at home in order to prepare for their recovery but had difficulty raising the energy to cope.

*Well, I had quite a high fever. Then it passed but came back; maybe it was a week later. I remember how exhausted I was when I was out shoveling snow, and I could not do anything; I was very short of breath for the smallest little thing. Then I got a high fever again.*

Suddenly, they fell ill again. The participants described that they became seriously ill this time, which frightened them more than at the onset of the disease. Still, at this point, the participants did not think they would become so critically ill that they would need intensive care and even less need respirator care. After contact with the healthcare provider, the participants were advised to go to the hospital. They were transported by their partner in their own car or ordered an ambulance. The memory of the time spent in the hospital before they were intubated was described as fuzzy, as they had difficulty breathing and felt weak.

*The cough was when I got lack of oxygen. When I made an effort, then it was worse, it was the lack of oxygen…in the evening they put me on a respirator, then I know nothing more.*

### A Living Nightmare

The participants described their experiences while critically ill as being in a nightmare from which they could not wake up. The nightmares they experienced were most evident during the most critical period in their illness when they were respirator-treated. Some participants, however, described also having the same kind of terrifying experiences before and after their stay in ICU.

*I already had such nasty dreams at home, really nasty dreams that I was welded into a car, and then I was thrown along the road, and it was so weird, really like that where I was drowning, and it was all sorts of things like that.*

The nightmares were terrifying for the participants; in them, their family got killed, there was a war going on, experiences of being hunted by big dark birds, having monsters, small children, and fire in the ventilation. There could be some connection to real events; for instance, those who had been transported by helicopters experienced helicopters in their dreams, and staff who had been dressed in yellow and white protective clothing and protective masks were experienced as strange figures, for instance, LEGO-like figures. Although participants felt they did not remember their stay in ICU, they provided details connected to ICU but mixed up with unreal events. There were experiences in which the staff was going to sell their organs, and one participant tried to push a rescue button on his arm so his wife would be able to rescue him. These nightmares were experienced in colors, felt completely real, and were impossible to wake up from. It was not until they were told what was real and when they could talk by phone to a family member who they thought was dead, that they realized what was real or unreal.

*There was a hole in the wall with cobras and crocodiles in it and a big spider, so at night, I thought: I can stand it for one night but not anymore.*

*I didn’t think they [relatives] were alive. I ended up in Paris on some COVID-19 ward; my partner found me there, suddenly we were in India. It was so hot, so disgusting to lie there, several thousands of people, everyone was very sick, people ran around with terrible breathing masks. Everyone had yellow-white striped coats, and they actually had these; somehow, I got the coats with me, they were in my nightmares, that everyone had such… The nightmares… If I close my eyes, I can remember, they got really stuck in my mind. I am very ill, I do not get any help, people think it is just as good that I die, and then all my loved ones die in strange and cruel ways. Most things take place in helicopters of various kinds.*

### Feeling Weak and Lonely

It was challenging for participants to become dependent on others to manage things they previously managed themselves. First, when they were ill at home, they became dependent on their family’s support. Thereafter, they became dependent on the healthcare staff before, during, and after their critical illness time. At the same time, the participants described a feeling of relief when staff took over, as they were too weak to take care of themselves. The participants felt gratitude for surviving COVID-19 and for the care they received, but did not want to go through anything similar again and they described that there were times when they thought about giving up and at the same time suppressed emotions to remain control.

*You are dependent on others; it is like being trapped in someone else’s body. It still is…*

*It was many times during the corona that I wanted to die. Had I been able to press an off button there and then, I would have smacked it with both hands immediately. Now the brain is like the brain is, so the longer time passes, the less hard I think it was, but I remember how terribly awful I thought it was. It was hard, damn how hard it was.*

It was frustrating for participants to be unable to say what they wanted to say and too weak to write it. After the stay in ICU, participants described trying to walk to the toilet, but their legs did not bear their weight. Using diapers and needing help from others to be washed were described as shameful. Feelings of weakness were described and worry about how to get their strength back. Worries about the future were commonly described.

*Of course, I was worried and sad, cried a lot. I got frustrated, felt like “Damn, should life be like this? Should I never be able to talk again?” Although I understood that when the respirator disappears, I should be able to talk again, I had a bit of a panic about it, that I could not make myself understood.*

Participants described feelings of loneliness, even though there could be staff around them. Being isolated, having trouble communicating, and not having relatives near increased the feeling of loneliness. For one participant, a pine tree outside the window became a friend and support during the critical illness. The pine tree could “tell” what weather and season it was, which was described as comforting.

*That pine, it was like my friend…The staff came in, they did what they were supposed to, and someone sat and talked to me at some point, held my hand when I was a little sad, it happened that I was (sad), so it has been such fantastic treatment of everyone. But still, you are very lonely anyway, and then I had my pine, and it was so weird. But yes, it was like it told me that today it is, oh, it has snowed today, and it was probably the weather there, April weather, it was a day that it was very snowy and so on.*

The participants missed having their close relatives present, especially when waking up during and after their ICU stay. They had been in contact with their relatives by phone before they were intubated and sent and received text messages they did not initially remember. Participants dreamed about their relatives in their dreams, and terrifying events happened to them. One participant filmed terrifying events with his phone to show his wife, but there was nothing that had been recorded. Talking to and seeing their relatives by phone for the first time after extubation was a touching, joyful moment when the participants realized their relatives were still alive. Their relatives had delivered special soft drink and papers to them at the hospital, even though they could not visit and had to travel a long way to the hospital. The participants felt guilt for their relatives and all their worry; they said they had been asleep for weeks, not knowing anything about what happened while their relatives were worried and isolated at home. They described their relatives as important to describe what had happened, and some had relatives who had written a diary for them at home during their illness.

*Yes, my wife has written a diary, everything she had talked to staff about, so she has written a diary, and I have read. I started from the back, and then it has not happened that I have read everything, but yes, then the status was oxygenation and such there, and it turns out that I have been so and so, and here and there and how many times a day they had turned me…and yes, she has noted all the stuff she got information about.*

### Discussion-Comprehensive Understanding

In this study, we suggest that the meaning of being critically ill in COVID-19 is that a frightening unreality becomes reality. Something terrible that was seen as unreal and something that could not happen suddenly became real and meant a struggle to survive. The question “Why did I become critically ill?” is recurrent among the participants, especially as they initially did not understand the severity of COVID-19. Toombs (2013) states that the onset of illness confronts one directly with one’s personal vulnerability. Loss of control is central to the experience of illness, especially when the illness is serious and unexpected, as was the case for the participants in this study. Initially, they saw it as something happening far away—affecting others—not themselves. They were, therefore, surprisingly hit by unpredictability when they became ill. According to Morse and Penrod (1999), when sudden events threaten the health and life of a person, they cease grasping the situation to make the incomprehensible more real. Morse and Johnson (1991) state that finding the reason for the illness can contribute to making sense of the event.

Despite their weakness, the participants in this study tried to manage daily tasks when they felt better. They started to focus on the future, “back-on-track” state, why the deterioration in the illness shook their existence. From Toombs’ (2013) writings, this can be understood as a loss of the familiar world where one can no longer assume that things will continue as they have in the past. Becoming critically ill in COVID-19 imposed physical and psychological restrictions in the participants' lives. It was a new, unknown illness, which, in turn, diminished predictability and forced the participants to face the unreal as real without clear explanations. This unpredictable state can be seen as enduring, which means the participants tried to maintain control through suppressing emotions (cf. Morse & Penrod, 1999).

Being critically ill felt like being in a nightmare participants could not wake up from; they repeatedly needed information about what was real and what was unreal. Kotfis, Roberson et al. (2020) suggest that the COVID-19 infection, the immune response of the body, the long-term respiratory treatment, and impaired respiratory efficiency during and after the COVID-19 infection are some of the medical reasons for the occurrence of nightmares or delirium during the ICU stay. Also, the isolation requirements have had an influence in many ways, and having these terrifying experiences in ICU may be one negative effect of the isolation (Kotfis, Roberson et al., 2020). The relatives’ presence is particularly important for patients who are confused (Engström, 2008; Magarey & McCutcheon, 2005), unable to communicate, or advocate for their interests (Kotfis, Williams Roberson et al., 2020). In Munro et al. (2017), patients who heard their relatives in pre-recorded voice messages had more delirium-free days. Participants in the present study experienced hallucinations and horrible experiences, and family-assisted reorientation interventions such as audio messages or the use of a digital technique where sound and image are put together might have helped. As soon as participants in the present study woke up, they appreciated talking to their relatives and knowing they were alive. This further stretches the importance of family to anchor patients in ICU and illuminates the possibility to use technological devices as important to minimize negative psychological effects due to restricted visitation for patients severely ill in COVID-19, also seen in Shaban et al. (2020).

Experiencing horrific nightmares in which death was described as a common theme, patients described a feeling of powerlessness and drifting from reality to unreality. It seems the nightmares, or hallucinations, included real events, like not having relatives, where participants thought they were dead, or for instance, the LEGO-like figures participants experienced with the yellow-white clothes, similar to the way staff actually looked with their face masks and yellow-white fatigues. Magarey and McCutcheon (2005) suggested the importance of providing constant reassurance and explaining everyday ICU happenings, which helps patients understand what they are experiencing. Bambi et al. (2020) described how personal protective equipment hinders communication with patients, for example, difficulties in hearing due to protective masks or the protective overalls that made the ICU staff indistinguishable. The consequence might be that it is difficult for patients to recognize who is standing at their bedside (Bambi et al., 2020). This, together with heavy sedation and neuromuscular blockade to improve transpulmonary pressure during respirator treatment and to be able to lie in the prone position for 16 hours or longer (Möhlenkamp & Thiele, 2020), makes it difficult for the ill person to understand what is happening, even if an explanation is given. Non-pharmacological interventions such as regular orientation are vitally important (Kotfis, Williams Roberson et al., 2020).

The participants’ independence was threatened by critical illness due to COVID-19. They felt weak and dependent on others to manage things they previously could manage themselves. According to Morse and Johnson (1991), a lack of understanding about what is happening to one’s body undermines a person’s sense of power and control, and the person must make sense of the illness experience before he or she is able to regain a sense of control. The person is no longer able to trust his or her abilities and therefore must rely on others for support. As the person regains a sense of independence, he or she is able to regain a sense of control (cf. Morse & Johnson, 1991). It is evident that besides being severely ill, patients face other stressful situations during their ICU admission. Patients who have been critically ill in ICU are vulnerable and have feelings of anxiety and loneliness as they face an uncertain recovery trajectory (Calkins et al., 2021). Loneliness is a feeling of being by oneself. The person has no choice and does not want to be in that condition (Bekhet et al., 2008). The findings in this study show that the participants felt lonely as they missed their relatives who had been close in their everyday life. Stek et al. (2005) show that loneliness leads to more pronounced motivational depletion and “giving up.” According to Rokach (1988), interpersonal isolation refers to the feeling of being emotionally, geographically, and socially alone. It consists of the absence of intimacy, perceived social alienation, and abandonment. The absence of intimacy is a disturbing feature of loneliness and refers to the lack of a close, intimate, and caring relationship. Absence of intimacy means missing a specific relationship, that is, a person who is no longer present, or the absence of any intimate relationship in one’s life (Rokach, 1988). In the present study, participants wanted to have relatives present at their bedside, especially when waking up, which was impossible because of restrictive visitation. For ICU patients, this might propagate a sense of isolation, ultimately contributing to disorientation and lack of awareness (Kotfis, Williams Roberson et al., 2020). The relatives’ presence is particularly important when patients are unable to communicate or advocate for their interests (Kotfis, Williams Roberson et al., 2020). Being given a diary (Engström et al., 2009; Strandberg et al., 2018) and attending a follow-up visit to the ICU are appreciated as measures that will allow people who have been mechanically ventilated to fill in the missing time (Engström et al., 2008, 2018; Ednell et al., 2017). Even though there were limited possibilities for relatives to be present, they wrote about the daily information they got about the ill person, something participants appreciated. Ricoeur (1976) stated that mutual understanding relies on sharing the same sphere of meaning. The effects the restrictive visitation might have on patients and their relatives and ways to maintain connection are unknown. There is a need for further studies in this area. There is also need for further investigation of patients’ and relatives’ perception of the quality of care during the ICU stay and about their daily lives after the patients’ critical illness. Recovery from COVID-19 seems to be long-lasting and as Rubin (2020) state, some patients become long haulers, with extensive restrictions in daily life even long after infection. Investigation from both the patients’ as well as the relatives' angles could provide specific knowledge of areas of strength and areas for improvement for further use in the quality work and when prioritizing care interventions during a pandemic.

### Methodological Considerations

When studying a phenomenon as in this study and aiming to try to understand what it means, it is important not to take theories, perspectives, or common sense as given. To see the phenomenon in a new way, it is crucial to be open and sensitive and not make definite what is indefinite (Dahlberg et al., 2008). As specialist nurses within intensive care and nursing researchers with clinical work experiences with critically ill people, we have tried to be aware of our pre-understanding. Two of us authors have worked in a COVID-19 ICU, which means that we know about the context of participants’ experiences. Based on purposive sampling, we sought participants who experienced critical illness with COVID-19 for which they needed ICU care and mechanical ventilation. The sample size is considered sufficient, as the interviews are long, informative, and contain data with depth and richness, which is seen as a strength. According to Sandelowski (1995), a sample size should be big enough to give variations in the narrated experiences but small enough to permit a deep analysis of the data. Further, one of the researchers (the first author) conducted all interviews, which also can be seen as a strength. Related to the pandemic, ten of the narrative interviews were conducted by telephone. Even though face-to-face interviews should be the golden standard, especially on the first interview occasion, Novick (2008) states that in qualitative research, telephone interviews can allow participants to relax and more easily disclose sensitive information.

The choice of method for analysis has been done according to the method’s ability to do justice to the real-life world. A phenomenological hermeneutic interpretation consists of an internal process of validation; the naïve understanding guides the structural analysis, which in turn validates or invalidates the naïve understanding (Lindseth & Norberg, 2004). All researchers participated in the process of moving back and forth between parts and the whole until the naïve understanding was validated by the structural analysis. In the comprehensive understanding, the whole text was read again, with the naïve understanding, the findings of the structural analysis, our pre-understanding, and the literature in mind. In common critical reflection, we aimed to broaden and deepen our understanding and reach a new interpreted whole (cf. Lindseth & Norberg, 2004). According to Ricoeur (1976), the interpretation must not only be probable but more probable than any other interpretation. In this study, the findings are based on the most probable interpretation we could reach. The findings in this study cannot be generalized but can be transferred to similar situations in another context (Polit & Beck, 2021).

## Conclusion

People who have been critically ill due to COVID-19 did not think they would get ill with this sudden, severe, and unexpected illness. They experienced terrible nightmares where their relatives have been killed, and their relatives were not allowed to be with them due to the virus, which meant they missed them both in their dreams and in reality. Gratefulness was described for surviving COVID-19. At the same time, participants described thoughts of not being able to imagine going through this again, as it was so hard and stressful. They described themselves as being healthy, independent, and strong and then suddenly becoming ill, dependent on others, and weak. They felt fear and loneliness, as a frightening unreality had become a reality. In other words, as Missel et al. (2021) state about the lived experiences of COVID-19 infection: “It’s not just a virus!”
